# Clinical characteristics of anti-myelin oligodendrocyte glycoprotein antibody among aquaporin-4 negative neuromyelitis optica spectrum disorders in Egyptian patients

**DOI:** 10.1038/s41598-024-83760-2

**Published:** 2025-01-09

**Authors:** Sara I. Taha, Salwa I. Bakr, Nermeen T. Fouad, Dina Zamzam, Yasmine A. Mohamed

**Affiliations:** 1 Department of Clinical Pathology, Faculty of Medicine, Ain Shams University, Abassia, Cairo, Egypt; 2 Department of Neurology, Faculty of Medicine, Ain Shams University, Cairo, Egypt

**Keywords:** Anti-myelin Oligodendrocyte Glycoprotein, Aquaporin-4, Disability, MOGAD, Neuromyelitis Optica Spectrum Disorder, Immunology, Neuroscience

## Abstract

Some patients with neuromyelitis optica spectrum disorder (NMOSD)-like symptoms test negative for anti-aquaporin-4 (anti-AQP4) antibodies. Among them, a subset has antibodies targeting myelin oligodendrocyte glycoprotein (MOG), a condition now termed MOG antibody-associated disease (MOGAD). MOGAD shares features with NMOSD, like optic neuritis and myelitis, but differs in pathophysiology, clinical presentation, imaging findings, and biomarkers. The present study investigated the prevalence of anti-myelin oligodendrocyte glycoprotein (anti-MOG) antibodies in anti-AQP4 seronegative Egyptian patients initially diagnosed with NMOSD and the link between their presence and clinical characteristics and disease-induced disability to gain insights into MOGAD. This pilot cross-sectional study included 40 anti-AQP4 antibody-negative patients initially diagnosed with NMOSD, six children and 34 adults. They were screened for anti-MOG antibodies by the indirect immunofluorescence cell-based assay. Of all included patients, only 7.5% (*n* = 3) were positive for anti-MOG antibodies and had significantly higher disability scores than seronegative patients (*p* = 0.021). The presence of anti-MOG antibodies was not significantly associated with age (*p* = 0.696), gender (*p* = 0.232), type of relapse (*p* = 0.488), number of attacks (*p* = 0.968), family history of consanguinity (*p* = 0.211), family history of autoimmune disease (*p* = 0.608), nor with smoking (*p* = 0.608). Detecting anti-MOG antibodies in anti-AQP4-negative NMOSD patients is essential for accurate diagnosis and personalized treatment, as MOGAD is now recognized as a separate clinical entity.

## Introduction

Neuromyelitis optica spectrum disorder (NMOSD) is a rare autoimmune demyelinating disorder of the CNS with a prevalence that rarely exceeds 5 per 100,000^1^. It causes disabling episodes of optic neuritis and transverse myelitis^2^, accompanied by astrocyte death, axonal loss, vascular proliferation, and perivascular lymphocytic infiltration^3^. It is characterized by longitudinally extensive (> 3 vertebral segments) spinal cord lesions and the absence of oligoclonal IgG bands (in about 70–85% of cases)^4^.

The discovery of anti-aquaporin-4 (anti-AQP4) antibodies has significantly advanced the understanding of NMOSD, differentiating it from other demyelinating diseases such as multiple sclerosis (MS)^5^. AQP4, the mammalian CNS’s most prevalent water channel, is highly expressed in the membrane of the astrocytic end-feet. Anti-AQP4 antibodies are pathogenic and primarily mediate humoral immune neuroinflammatory responses^6^, leading to high complement activation^7^. Despite the highly sensitive and specific assays for anti-AQP4 antibodies, up to 40% of NMOSD patients do not have these antibodies at initial presentation and during the disease course^8^.

Myelin oligodendrocyte glycoprotein (MOG), a member of the immunoglobulin superfamily, is expressed on the outer lamella of the myelin sheath and oligodendrocytes, making it more likely to be immunogenic than other CNS myelin proteins^2,9^. Anti-MOG antibodies synergize both encephalitogenic T-cell and antibody-mediated humoral demyelinating responses^10^. Therefore, anti-MOG antibodies are associated with oligodendropathy, while anti-AQP4 antibodies are associated with astrocytopathy^8^.

Recently, anti-MOG antibodies have emerged as a significant biomarker for distinguishing subsets of demyelinating diseases. Studies show that a subset of NMOSD patients, who are anti-AQP4-negative, test positive for anti-MOG antibodies. These patients are now described as having MOG-associated disease (MOGAD), exhibiting unique clinical, biological, and immunological profiles and distinct treatment responses compared to classic NMOSD patients^2,11–14^. MOGAD patients usually present at a younger age and have a milder disease with a lower relapse rate and better recovery^11–14^.

The detection of anti-MOG antibodies in anti-AQP4-negative NMOSD patients is therefore crucial for accurate diagnosis and tailored therapeutic strategies.

The current study aims to investigate the prevalence of anti-MOG antibodies among anti-AQP4 seronegative Egyptian patients initially diagnosed with NMOSD and to assess the association between these antibodies and the clinical features and disease-induced impairment to gain insights into the characteristics of MOGAD.

## Materials and methods

### Study design and subjects

This pilot cross-sectional study included 40 Egyptian patients, 6 children (age: <18 years old) and 34 adults (age: ≥18 years old); of them, 35% (*n* = 14) were males, and 65% (*n* = 26) were females, initially diagnosed with NMOSD by clinical picture and radiological findings according to the 2015 international consensus diagnostic criteria of NMOSD^15^. They were recruited from the outpatient Neurology Clinic at Ain Shams University Hospitals, Cairo, Egypt. All included patients were seronegative for anti-AQP4 antibody by a cell-based assay (CBA). MS patients and those who received corticosteroids within a month before the study were excluded. The study was conducted following the ethical guidelines for research defined in the Declaration of Helsinki. This study was carried out after the approval of the ethical committee of Ain Shams University, Faculty of Medicine (FMASU MS176/2019). Before participating in this study, all subjects or their primary relatives conferred a written informed consent form. All collected data were kept private and confidential and were solely utilized for the study’s purposes.

### Clinical assessment

The neurologist examined the disability status of all participants using the Expanded Disability Status Scale (EDSS), which offers a scale ranging from 0 to 10 in 0.5-unit increments, with higher scores indicating a greater degree of disability. People with a high degree of ambulatory ability are classified as levels 1.0 to 4.5, whereas those with a loss of ambulatory capacity are classified as levels 5.0 to 9.5^16^.

### Sample collection and anti-MOG antibody analysis

**Fig. 1:**
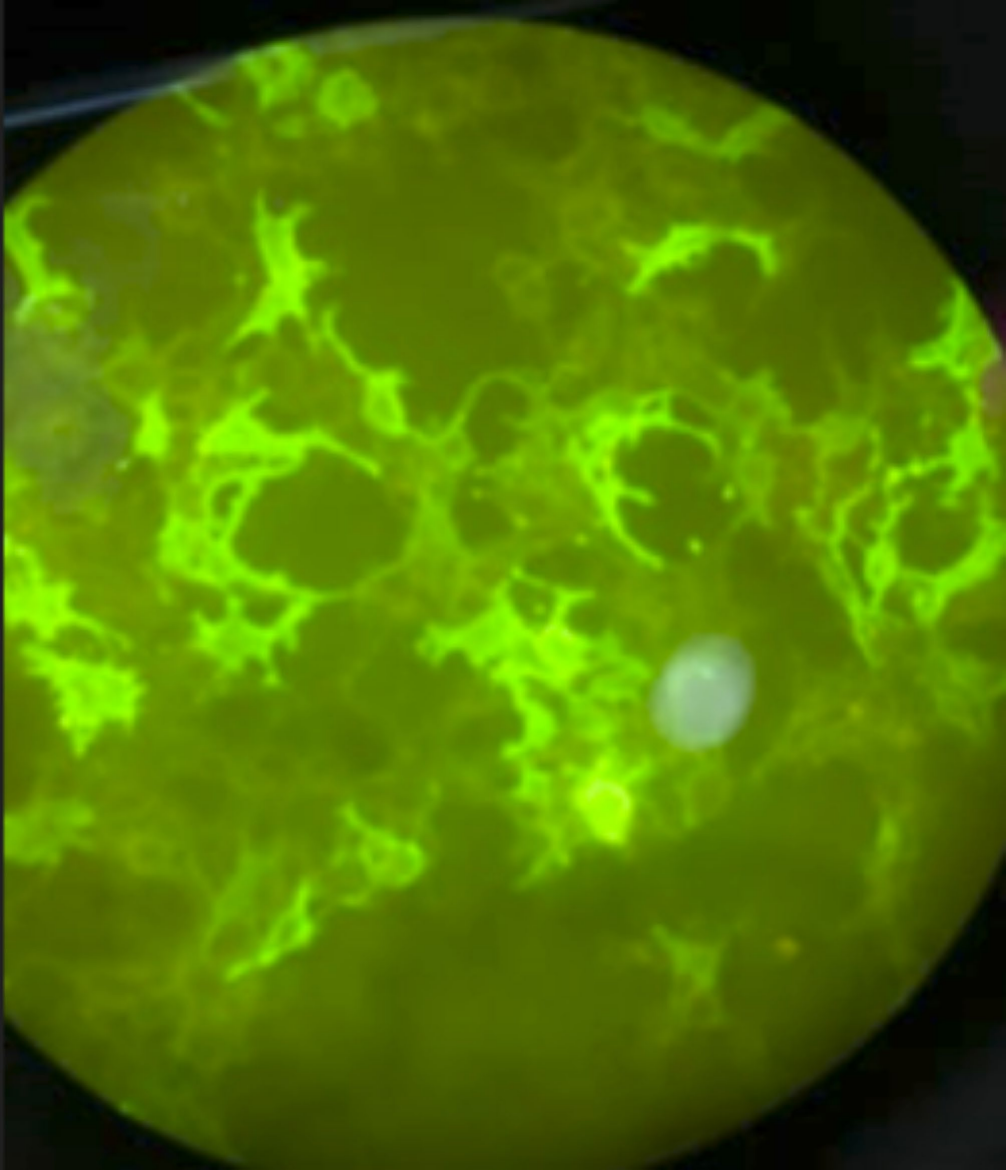
Positive anti-MOG antibody by indirect immunofluorescence technique.

From each participant, 3 ml venous blood was collected by aseptic venipuncture into a serum separation vacutainer tube. Blood samples were allowed to clot completely and centrifuged at 3000×g for 10 min. Separated sera were collected and stored in the freezer (-80°C) until analysis. Anti-MOG IgG antibody detection was done using indirect immunofluorescence CBA (EUROIMMUN Medizinische Labordiagnostika AG, Lübeck, Germany, order no.: FB 1156-1005-50) according to manufacturer’s instructions. The slides were assessed with a fluorescence microscope (Olympus CX33 Biological Microscope, Tokyo, Japan) using a 40x magnification power lens; positive reactions produced a flat, smooth to coarse-granular fluorescence of the cell with an accent of the cell membrane. The area of the cell nucleus was only slightly stained. Figure 1.

### Statistical analysis

We used the Statistical Package for Special Sciences (SPSS) software computer program version “V. 23.0” (IBM Corp., USA, 2015) for data analysis. Quantitative nonparametric data were described using median and 25th – 75th percentile, while quantitative parametric data were described using mean ± standard deviation (SD). The description of qualitative data was presented as numbers and percentages. For comparison, the Mann-Whitney and the Chi-square tests were used. The significance level was set at *p*-value < 0.05.

## Results

A total of 40 anti-AQP4 antibody seronegative patients initially diagnosed with NMOSD were included in this cross-sectional study. The patients’ mean (± SD) age in the present study was 29.5 (± 12.8) years. Only 7.5% (*n* = 3) patients had a positive family history (FH) (of second-degree type) of autoimmune disease, and (32.5%) (*n* = 13) patients had a positive FH of consanguinity (of 3rd-degree type); however, none of all participants had a positive FH of MS.

Since the onset of the disease, 32.5% (*n* = 13) of all included subjects experienced relapse, with the majority 53.85% (*n* = 7) having only one attack. 69.23% (*n* = 6) of the relapsed patients had sensory (optic nerve) manifestations. During the attack, 61.54% (*n* = 8) of the patients received pulse steroids solely, and 60% (*n* = 24) of all included patients were maintained on vitamins and iron supplementation. Table 1.

**Table 1:** Demographic and clinical characteristics of the studied patients.

Gender n., (%)	Male	14 (35.0%)
	Female	26 (65.0%)
Age (years)	Range	10–62
	Mean ± SD	29.50 ± 12.81
Risk factors n., (%)	Smoking	3 (7.50%)
	FH of MS	0 (0.00%)
	FH of consanguinity	13 (32.50%)
	FH of autoimmune disease	3 (7.50%)
Relapsed patients n., (%)		13 (32.50%)
Number of attacks n., (%)	One	7 (53.85%)
	Two	2 (15.38%)
	Four	1(7.69%)
	Five	1(7.69%)
	Six	1(7.69%)
	Eight	1(7.69%)
Type of attacks n., (%)	Cranial nerves (optic)	9 (69.23%)
	Motor	4 (30.77%)
Treatment during attack n., (%)	Pulse steroids	8 (61.54%)
	IVIG	4 (30.77%)
	Mixed	1(7.69%)
Maintenance treatment n., (%)	Methotrexate	2 (5.00%)
	Vitamins &Iron	24 (60.00%)
	Oral steroids	3 (7.50%)
	Azathioprine	6 (15.00%)
	MabThera™/Rituximab	5 (12.50%)

F.H.: Family history; IVIG: Intravenous immunoglobulin; MS: Multiple sclerosis.

**Fig. 2:**
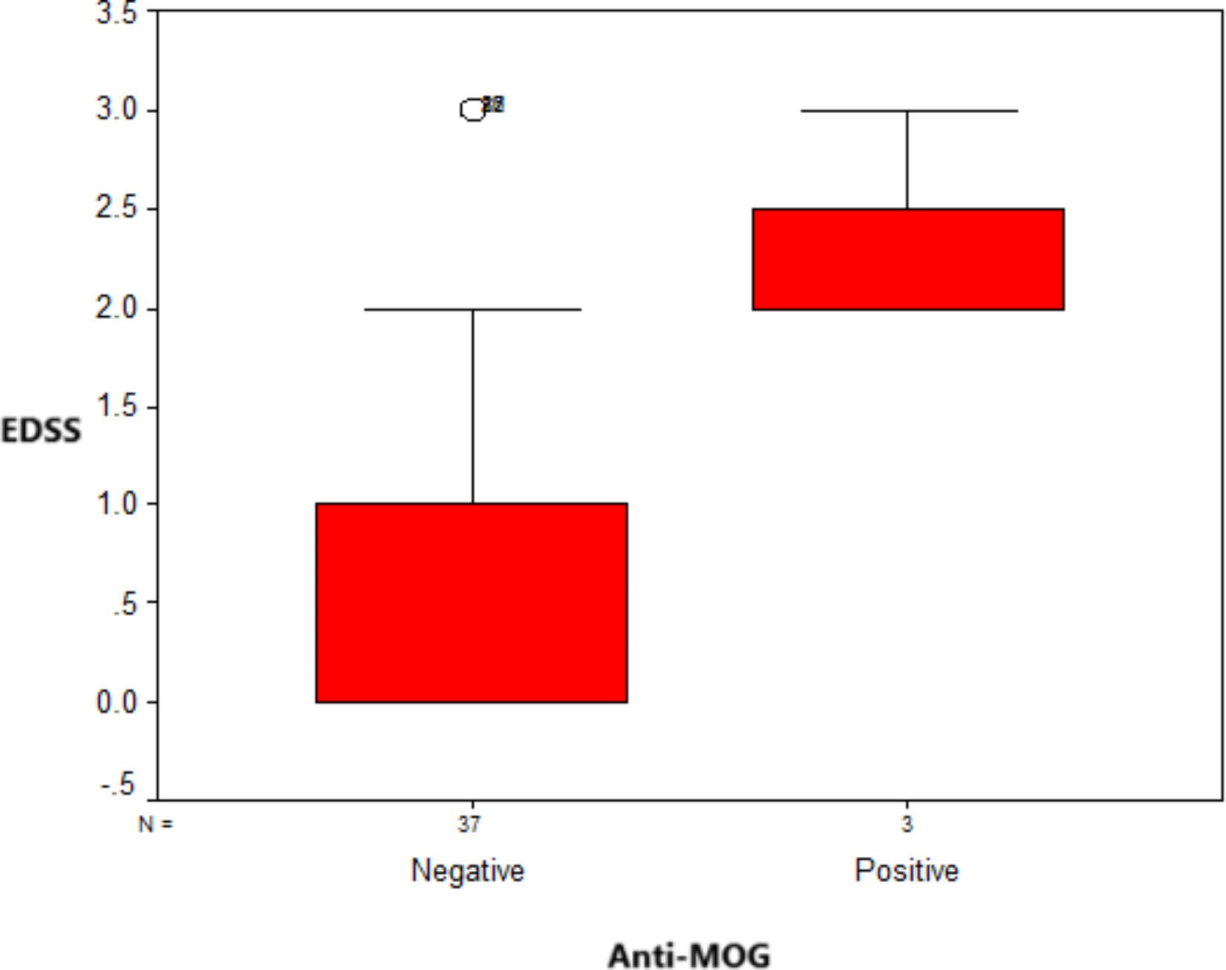
Box plot comparison between the anti-MOG seropositive and negative patients regarding their EDSS scores (Median 25th – 75th percentile 2 (2–3) vs. 0 (0–3); *p* = 0.021).

Of all included patients, only 7.5% (*n* = 3) were positive for anti-MOG antibodies. Seropositive patients had significantly higher EDSS scores compared to seronegative patients (Median 25th – 75th percentile 2 (2–3) vs. 0 (0–3); *p* = 0.021)). Figure 2.

However, the presence of anti-MOG antibodies was not significantly associated with age (*p* = 0.696), gender (*p* = 0.232), type of relapse (*p* = 0.488), number of attacks (*p* = 0.968), FH of consanguinity (*p* = 0.211), FH of autoimmune disease (*p* = 0.608), nor with smoking (*p* = 0.608). Table 2.

**Table 2:** Comparative statistics of anti-MOG with demographic and clinical parameters.

		Anti-MOG						
		Negative (n = 37)			Positive (n = 3)			*P*-value
Age (years)	Range	10	-	62	12	-	40	0.696
	Mean ± SD	29.73	±	12.89	26.66	±	14.04	
		N		(%)	N		(%)	*P*-value
Gender	Male	12		(32.43)	3		(100)	0.232
	Female	25		(67.57)	0		(0)	
Risk factors	Smoking	3		(8.11)	0		(0.00)	0.608
	FH of MS	0		(0.00)	0		(0.00)	-
	FH of Consanguinity	13		(35.14)	0		(0.00)	0.211
	FH of Autoimmune disease	3		(8.11)	0		(0.00)	0.608
Relapses	No	25		(67.57)	2		(66.67)	0.974
	Yes	12		(32.43)	1		(33.33)	
Number of attacks	One	6		(50.00)	1		(100.0)0)	0.968
	Two	2		(16.67)	0		(0.00)	
	Four	1		(8.33)	0		(0.00)	
	Five	1		(8.33)	0		(0.00)	
	Six	1		(8.33)	0		(0.00)	
	Eight	1		(8.33)	0		(0.00)	
Type of attacks	Cranial nerves(optic)	8		(66.67)	1		(100.00)	0.488
	Motor	4		(33.33)	0		(0.00)	
Treatment during attack	Pulse steroids	7		(58.33)	1		(100.00)	0.713
	IVIG	4		(33.33)	0		(0.00)	
	Mixed	1		(8.33)	0		(0.00)	
Maintenance treatment	Methotrexate	2		(5.41)	0		(0.00)	0.826
	Vitamins &Iron	21		(56.76)	3		(100.00)	
	Oral steroids	3		(8.11)	0		(0.00)	
	Azathioprine	6		(16.22)	0		(0.00)	
	MabThera™*/*Rituximab	5		(13.51)	0		(0.00)	

F.H.: Family history; IVIG: Intravenous immunoglobulin; MS: Multiple sclerosis.

Significance was set at *p* < 0.05.

## Discussion

Previous studies reported that a minority of negative anti-AQP4 NMOSD patients have anti-MOG antibodies and show different pathogenesis and outcome of the disease^17–19^. However, MOGAD is much broader than NMOSD, and only a minority of MOGAD patients fulfil NMOSD criteria^11–14^. Early and accurate diagnosis allows for proper acute and long-term immunosuppressive therapy and minimizing disability^17^. Data on MOGAD in North Africa are sparse, and the significance and prevalence of MOGAD still need to be determined^20,21,11–14^.

In the current study, only 7.5% (3/40) of our included patients tested positive for the anti-MOG antibody (1 child and 2 adults). All of them were males. Similarly, a study by Sato et al.^22^ revealed that among their 215 patients with NMOSD, 7.4% (16/215) were positive for anti-MOG antibodies, and this percentage increased to 21.1% (16/76) when anti-AQP4 negative NMOSD were considered. Their study also reported male predominance among NMOSD patients with anti-MOG reactivity (female to male ratio: 0.6:1.0 [6/10]). Similarly, Kitley et al.^7^ reported a male predominance of 56% among anti-MOG positive patients.

Partially similar to our results, a study conducted on 42 Algerian patients with optic neuritis and/or myelitis by Bouzar and colleagues in 2017^20^ revealed that 7.1% (3/42) of their patients were positive for anti-MOG antibodies and negative for anti-AQP4 antibodies. However, all antibody-positive patients were women.

Comparably, in a study in Taiwan by Hsu et al. in 2021^18^, which tested 22 female patients with NMOSD for anti-AQP4 and anti-MOG antibodies, serum anti-AQP4 antibodies were found in 86% of their patients, while anti-MOG antibodies were found in one of the three (33.3%) of anti-AQP4 antibody-negative patients (4.5% of all the included patients in the study). Similarly, a cohort study in England by Kitley et al.^7^ has demonstrated that 34% of anti-AQP4 seronegative NMOSD patients tested positive for anti-MOG antibodies. Moreover, Ramanathan et al.^23^, Pröbstel et al.^24^, and Yan et al.^25^ reported an incidence rate of anti-MOG antibody among anti-AQP4 negative NMOSD patients of 39.1% (9/23), 23.5% (4/17) and 30.4% (14/46) respectively.

In the current study, 16.6% (1/6) of all children and 5.88% (2/34) of the adults tested positive for anti-MOG antibodies. The age range of our anti-MOG antibodies seropositive patients was 12–40 years. Similarly, Brill et al. in 2021^26^ stated that MOGAD can occur at any stage of life but has a significantly higher prevalence in children. They tested 683 patients (121 children and 447 adults) with demyelinating diseases for anti-MOG antibodies. They found that the age range of anti-MOG antibodies seropositive patients was 1–66 years, with significantly increased prevalence in children (19% vs. 6.7% in adults).

On the other hand, a multicenter study in France compared the clinical features and anti-MOG antibody dynamics in 98 children and 268 adults with MOGAD and reported that MOGAD were more frequent in adults than in children^27^. The discrepancy in results could be due to different subsets of patients in the present study (anti-MOG positive anti-AQP4 negative NMOSD patients) and those of Cobo-Calvo and his colleagues^27^, which included all anti-MOG positive demyelinating diseases.

The current study found no significant association between anti-MOG seropositivity with neither smoking nor positive FH of autoimmune diseases and neurological disorders. On the contrary, Messina et al. in 2021^28^ stated that among the 70 seropositive anti-MOG antibodies NMOSD patients included, smoking was significantly associated with worse disease outcomes but not relapse risk. Moreover, to the best of our knowledge, no previous studies have discussed the association between FH and anti-MOG antibodies seropositivity, a point that needs further investigation.

MOGAD can be either monophasic or relapsing and generally leads to less disability compared to NMOSD, although there is notable variability in outcomes between patients^12,29^. Uzawa et al. in 2024 reported that the initial attack accounts for over 70% of the disability associated with MOGAD^29^. However, the median EDSS score in anti-MOG seropositive individuals in the present study was 2.0 (two patients with EDSS = 2 and one with EDSS = 3). It was significantly higher than the median EDSS scores in seronegative patients (*p*-value = 0.021), reflecting the higher degree of disability among anti-MOG-positive patients. Alshamrani et al.^21^ published a case series in which they assessed the records of nine anti-MOG seropositive people and reported that the median EDSS score for these patients was 3.0 (range, 2.0–4.0).

The effectiveness of anti-MOG antibody surveillance in predicting attacks is still under debate, and the dynamics of these antibodies are still unknown. In the current study, only one (33.33%) of the anti-MOG seropositive patients had one attack of relapse in the form of optic nerve affection. In 2019, Lana-Peixoto and colleagues^19^ reported that most patients with MOGAD had a relapsing course and monophasic disease incidence declined with follow-up. Similarly, Jarius and colleagues in 2016^30^ reported that relapses occurred in 93% of anti-MOG positive anti-AQP4 negative NMOSD patients with disease duration ≥ 8 years and that 88% of the relapses were in the form of optic neuritis. In addition, Akaishi et al. in 2022^31^, reported that approximately 50% of the patients with MOGAD experienced relapses in the first 10 years. Many other studies reported optic neuritis as the first clinical presentation and the most common relapse type^26,32–34^.

Patients with MOGAD typically respond to drug treatment but relapse after prednisone discontinuation or quick tapering^35,36^. In that context, one of our anti-MOG antibody-positive children patients had a sensory relapse shortly after his first attack, presumably due to inadequate treatment in his first assault when he was misdiagnosed as having encephalitis and only received antibiotics with partial recovery but encountered remarkable recovery after receiving five doses of pulse steroids. Carnero Contentti et al. in 2021^37^ reported that the clinical manifestations of MOGAD vary by age. Younger children predominantly present with acute disseminated encephalomyelitis (ADEM) or ADEM-like symptoms, while older children and adults more commonly experience optic neuritis and transverse myelitis^37^.

The discrepancies observed between our study and others may stem from various contributing factors, including differences in sample sizes, ethnic backgrounds, disease stages, and the methodologies used for anti-MOG detection (such as cell-based assays (CBA), enzyme-linked immunosorbent assay (ELISA), and immunohistochemistry (IHC)). Additionally, variations in follow-up durations could also play a role in these differences.

Our research contributes significantly to unraveling the distinctive nature of anti-MOG positivity in anti-AQP4-negative NMOSD, specifically in the context of MOGAD. However, it has some flaws that need to be addressed. For instance, the sample size is relatively small due to the disease’s rarity. Second, there is no data about the duration and course of the disease and the timing of blood sampling concerning attacks. Therefore, conducting multicenter prospective studies with larger sample sizes and extended follow-up periods is crucial for further elucidating the distinctions between classic NMOSD and MOGAD.

## Conclusions

The detection of anti-MOG antibodies in anti-AQP4-negative NMOSD patients is crucial for accurate diagnosis and tailored therapeutic strategies, as MOGAD is increasingly recognized as a separate clinical entity with specific pathophysiological mechanisms and prognostic implications.

## Data Availability

The datasets used and/or analysed during the current study available from the corresponding author on reasonable request.
